# Particulated, Extracted Human Teeth Characterization by SEM–EDX Evaluation as a Biomaterial for Socket Preservation: An In Vitro Study

**DOI:** 10.3390/ma12030380

**Published:** 2019-01-25

**Authors:** José Luis Calvo-Guirado, Alvaro Ballester Montilla, Piedad N De Aza, Manuel Fernández-Domínguez, Sergio Alexandre Gehrke, Pilar Cegarra-Del Pino, Lanka Mahesh, André Antonio Pelegrine, Juan Manuel Aragoneses, José Eduardo Maté-Sánchez de Val

**Affiliations:** 1 Department of Oral and Implant Surgery, Faculty of Health Sciences, Universidad Católica San Antonio de Murcia, 30002 Murcia, Spain; jlcalvo@ucam.edu; 2 International Dentistry Research Cathedra, Universidad Católica San Antonio de Murcia, 30002 Murcia, Spain; aballestermontilla@hotmail.com (A.B.M.); pilarcedepi@gmail.com (P.C.-D.P.); 3 Institute of Bioengineering, Miguel Hernández University, 03203 Elche-Alicante, Spain; piedad@umh.es; 4 Department of Translational Medicine, CEU San Pablo University, 28223 Madrid, Spain; 5 Biotecnos Research Center—Tecnología e Ciencia, Ltd., Montevideo 11800, Uruguay; sergio.gehrke@hotmail.com; 6 Implantologist, Private Practice, New Delhi 110002, India; drlanka.mahesh@gmail.com; 7 Faculdade São Leopoldo Mandic, Instituto de Pesquisas São Leopoldo Mandic, São Paulo 13024-530, Brazil; andre.pelegrine@slmandic.edu.br; 8 Department of Dental Research in Universidad Francisco Henríquez y Carvajal, 10107 Santo Domingo, Dominican Republic; jmaragoneses@gmail.com; 9 Department of Oral and Implant Surgery, Faculty of Health Sciences, Universidad Católica San Antonio de Murcia, 30107 Murcia, Spain; jemate@ucam.edu

**Keywords:** smart dentin grinder, autogenous particulate dentin graft, tooth graft, ground teeth, human teeth, bone grafts, autologous graft

## Abstract

The aim of the study was to evaluate the chemical composition of crushed, extracted human teeth and the quantity of biomaterial that can be obtained from this process. A total of 100 human teeth, extracted due to trauma, decay, or periodontal disease, were analyzed. After extraction, all the teeth were classified, measured, and weighed on a microscale. The human teeth were crushed immediately using the Smart Dentin Grinder machine (KometaBio Inc., Cresskill, NJ, USA), a device specially designed for this procedure. The human tooth particles obtained were of 300–1200 microns, obtained by sieving through a special sorting filter, which divided the material into two compartments. The crushed teeth were weighed on a microscale, and scanning electron microscopy (SEM) evaluation was performed. After processing, 0.25 gr of human teeth produced 1.0 cc of biomaterial. Significant differences in tooth weight were found between the first and second upper molars compared with the lower molars. The chemical composition of the particulate was clearly similar to natural bone. Scanning electron microscopy–energy dispersive X-ray (SEM–EDX) analysis of the tooth particles obtained mean results of Ca% 23.42 ± 0.34 and P% 9.51 ± 0.11. Pore size distribution curves expressed the interparticle pore range as one small peak at 0.0053 µm. This result is in accordance with helium gas pycnometer findings; the augmented porosity corresponded to interparticle spaces and only 2.533% corresponded to intraparticle porosity. Autogenous tooth particulate biomaterial made from human extracted teeth may be considered a potential material for bone regeneration due to its chemical composition and the quantity obtained. After grinding the teeth, the resulting material increases in quantity by up to three times its original volume, such that two extracted mandibular lateral incisors teeth will provide a sufficient amount of material to fill four empty mandibular alveoli. The tooth particles present intra and extra pores up to 44.48% after pycnometer evaluation in order to increase the blood supply and support slow resorption of the grafted material, which supports healing and replacement resorption to achieve lamellar bone. After SEM–EDX evaluation, it appears that calcium and phosphates are still present within the collagen components even after the particle cleaning procedures that are conducted before use.

## 1. Introduction

In recent years, the physicochemical properties of biomaterials have been analyzed extensively to identify characteristics that will maximize the clinical outcomes of bone defect repair. In this context, two characteristics—grain size and the biomaterial’s composition—directly influence the biomaterial’s resorption activity and the speed of resorption [1].

A bone replacement material must have “bimodal” behavior, which, in the early stages of differentiation, allows osteoblasts to build bridges between grains of different sizes and integrate with other osteoblasts, supporting both proliferation and differentiation. New bone formation is stimulated by the activation of mesenchymal stem cells on the rough surfaces of biomaterials [2,3,4]. The ultimate goal is the union of completely differentiated osteoblasts, which will support the production of the bone matrix. This requires a bone replacement material with a porous structure including macropores, micropores, and nanopores [5,6]. In terms of roughness and external porosity, the surface of the bone replacement material’s particles will directly influence the attachment of solvents to the surface of the biomaterial, allowing advanced cell colonization and the process of biomaterial remodeling to commence.

The presence of macropores and micropores in the particles of the graft biomaterial has been shown to be a very important criterion, allowing blood vessels to enter and favoring bone growth through osteoconduction within the pores. The structural properties and the physical and chemical characteristics of composite ceramics have been seen to affect their behavior in vivo, whether dependently or independently, whereby the outcome will depend on the case’s individual bone repair parameters. Synthetic scaffolds can be used in regenerative and reconstructive surgery to treat bone defects. Biomaterials consisting of collagen and ceramic material are typically evaluated in terms of the proportions of liquid, collagen, and hydroxyapatite they contain. Porcine hydroxyapatite (HA) has lower crystallinity due to the presence of collagen in its composition. Changing the size, porosity, and crystallinity of each HA-based bone substitute material will influence the integration of the biomaterial within the implantation site and new bone formation [7,8]. To allow tissue penetration into the pores (and thus bone repair), they must be greater than 100 µm [9,10,11,12,13].

The most commonly used biomaterials are bioceramics based on calcium phosphate (Ca-P). The Ca-Ps have a composition and structure highly similar to the bone mineral phase, which presents osteoconductive properties and thus stimulates bone formation. Among the various materials assayed in recent years, tricalcium phosphate (TCP) has shown promising results in animal experiments and clinical studies [14,15,16].

At least one case series and several animal studies have reported promising results from a technique in which extraction sockets were augmented with autologous, particulate, mineralized dentin placed immediately after tooth extraction [17,18,19]. Although the supply of human teeth is, in fact, limited, when an extraction takes place, the tooth is naturally available and should be used to correct the damage caused by the extraction and subsequent lack of function, which leads to extensive resorption. To perform this procedure, the “Smart Dentin Grinder” ^TM^ machine was specially designed to crush, grind, and classify extracted teeth into different size particles. A special Dentin chemical cleanser is applied for 5 min to eliminate bacteria from the tooth, and right after, the tooth is washed with PBS two times. This novel procedure can be performed with any extracted teeth. Technically speaking, an autologous material can be returned to its donor without any treatment. However, in the case of the protocol that we have followed, there are multiple steps where strong disinfectant agents are used that are very effective in removing any bacteria/virus and many other biohazards that might be present.

Although this is true for allografts, this is not the case for autografts. Using our own biology in order to treat ourselves is not subject to ethical considerations.

The aim of this study was to determine the chemical composition and the amount of biomaterial obtained from crushed human teeth in order to fill empty alveolus with material from the manufacturer‘s protocol. 

## 2. Materials and Methods 

The study protocol was approved by the Catholic University of Murcia Ethics Committee (UCAM; registration number 6781; 21-07-2017).

Human teeth were extracted from 50 patients aged between 36 and 65 years, who received no financial compensation. All the patients signed informed consent forms to donate their teeth for use in the study. The teeth were extracted because of trauma, decay, or periodontal disease that had caused damage to one or two teeth in the upper maxilla and/or mandible. A total of 100 teeth were collected from 50 donors. The teeth were cleaned using straight fissure carbide burs, trimming the periodontal ligament, and dried with an air syringe. Each tooth was immediately classified, measured, and weighed. All the teeth were stored in separate sterile crystal containers at room temperature for 1–3 months, 1 per donor, labeling each container with the donor’s details and the characteristics of the teeth (type, weight, dimensions). 

**Figure 1:**
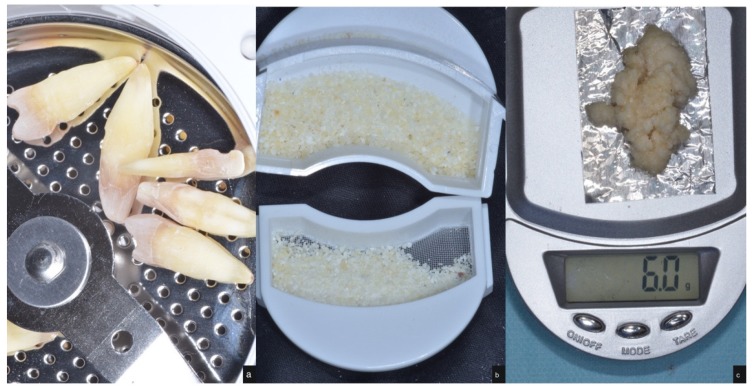
(**a**) Human teeth inside the Smart Dentin Grinder chamber; (**b**) Upper and lower compartment of different sized particles ranging from 300 to 1200 microns; (**c**) grounded teeth being weighed.

**Figure 2:**
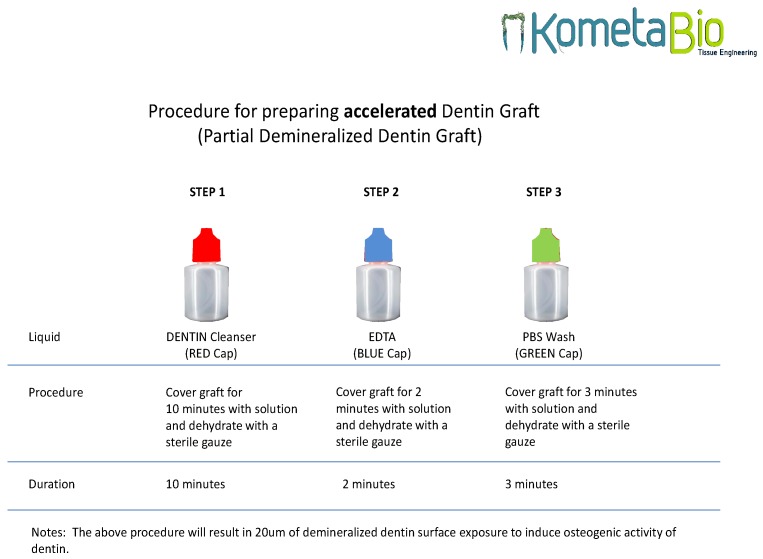
The manufacturer’s protocol for grinding teeth.

After being cleaned and dried, the teeth were immediately crushed using the “Smart Dentin Grinder” device (KometaBio Inc., Cresskill, NJ, USA). The idea was to process an autologous dentin graft as a replacement for autologous bone harvesting. By doing so, we can preserve the tooth in the form of a particulate without diminishing the bioactive properties of dentin, a plethora of BMPs (bone morphogenic proteins) and growth factors, therefore leveraging it as a biocompatible, bioactive, bio-inert graft. Using dentin for non-autologous purposes or alternatively as an allograft, which requires extensive processing, is certainly not efficient and is not part of the current study’s parameters. Autologous bone is still considered the gold standard for grafting. Autologous dentin not only has the same effects as autologous bone, we argue that, due to its inert and strong scaffold of dense HA, it is, in effect, better than autologous bone. The tooth particles were sized at 300–1200 microns, obtained by sieving the particles into two different compartments (Figure 1). The tooth particulate was then immersed in a basic alcohol cleanser in a sterile container for 10 min to dissolve all organic waste and bacteria. Afterward, the teeth particles were placed in ethylenediaminetetra-acetic acid (EDTA) for 2 min for partial demineralization and then washed with sterile saline for 3 min (Figure 2). Virus and fungi are all eliminated using the dentin cleanser that is part of the protocol. The dentin cleanser is a strong alkali (sodium hydroxide and ethanol combination) that is very effective in removing all bacteria, virus, and fungi. As for prions, we are not sure whether the dentin cleanser is able to remove all prions, but, again, these are the patient’s own prions, because this is an autologous graft.

**Figure 3:**
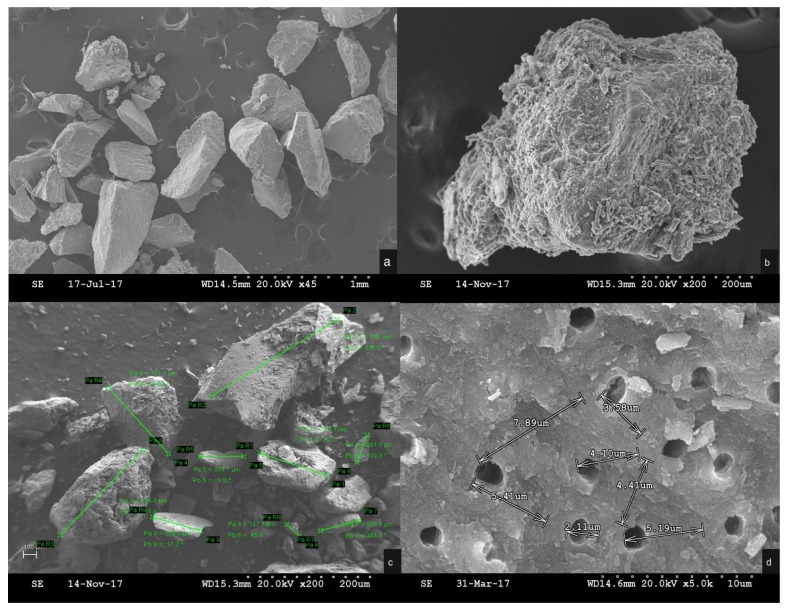
(**a**) Scanning electron microscopy of teeth particles at 1-mm magnification; (**b**) augmented evaluation of collagenized tooth particles at 200 microns; (**c**) particle measurements at 200 microns; and (**d**) dentin tube measurements at 10 microns.

**Figure 4:**
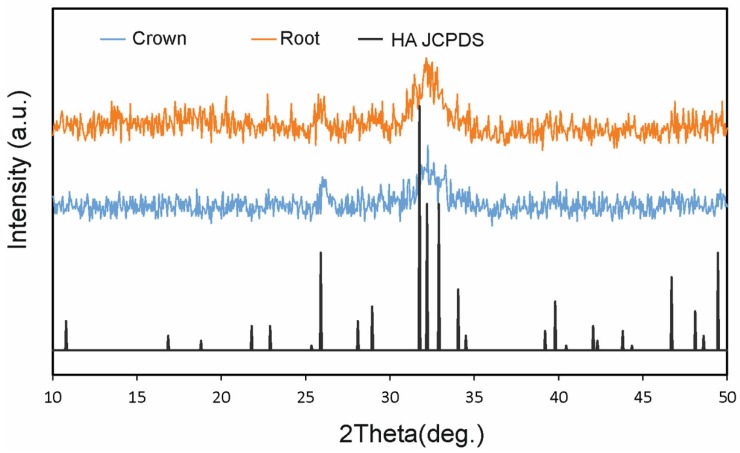
The diffractograms of the samples were compared with data from the Joint Committee on Powder Diffraction Standards (JCPDS) database.

The ground tooth material was analyzed by scanning electron microscopy (SEM) to evaluate its characteristics (Figure 3). For the SEM study, the particulate samples were placed in liquid nitrogen for approximately 2 min. The particles were coated with a carbon film (BalTec CED 030; BalTec, Balzers, Liechtenstein) for SEM analysis at ×10 magnification. The resolution was 0.8nm @ 15KV; 1.4nm @ 1 KV; 0.6 nm @ 30KV (STEM mode); 3.0@ 20 kV at 10 nA; and WD 8.5 nm using a Gemini II Electron Optics (Carl Zeiss Microscopy Gmbh, Jena, Germany), which is fitted with detectors for secondary electrons and backscattered electrons in order to allow for exploration of the different biological processes involved in tissue healing and to identify morphological changes in the cellular components of different materials. Mineralogical analysis of the material was performed by X-ray diffraction (XRD). XRD patterns were obtained using a Bruker AXS D8-ADVANCE X-ray Diffractometer (Karlsruhe, Germany) applying CuK1 radiation (0.15418 nm) and a second curved graphite monochromator. Diffractograms of the samples were compared with data from the Joint Committee on Powder Diffraction Standards (JCPDS) database (Figure 4).

The samples’ porosity and pore size distribution were analyzed by mercury porosimetry using an automatic pore size analyzer (Poremaster-60 GT, Quantachrome Instruments, Boyton Beach, FL, USA) within a 6.215–411,475.500 KPa pressure range, corresponding to a pore diameter range of 236,641.05–3.57 nm. A total of 3 particulate samples (~0.47 g) were analyzed using this technique. An additional sample was also used in every case if the measured values for porosity differed by more than 5%. Helium gas pycnometry (Quantachrome Instruments, Boyton Beach, FL, USA) was used to determine the particle’s real density (sample mass/volume of the solid), excluding empty spaces.

### Statistical Analysis

Statistical analysis was performed using PASW Statistics v.18.0.0 software (SPSS). A descriptive test of a mean and standard deviation of each human tooth length, width, and weight was conducted. One-way ANOVA was applied for the comparison of the means, assuming a level of significance of 95% (*p* < 0.05). 

## 3. Results

Human upper central incisors measured 6.5 ± 0.2 mm in length, 1.2 ± 0.6 mm in width, and weighed 1.3 ± 0.9 gr, while first mandibular molars measured 6.9 ± 0.2 mm in length, 2.1 ± 0.7 mm in width, and weighed 2.2 ± 1.1. These data show the significant differences between central incisors and first molars, which presented twice the width and weight of the incisors. Table 1 shows the mean tooth dimensions obtained for each type of tooth.

Figure 3 shows the X-ray diffraction (XRD) patterns of central incisor tooth particles. XRD patterns are associated with the biomaterial’s chemical composition. The crushed tooth particles presented high crystallinity (Figure 4). 

A human extracted tooth weighing 0.25 gr produced at least 1.0 cc of particulate (Table 2).

Analyzing the material by mercury porosimetry, two kinds of spaces were identified: those that correspond to empty spaces between particles (commonly designated as “interstices” or “interparticle” spaces) and those that correspond to the spaces within the particles themselves (known as “pores” or “intraparticle” spaces). The results obtained for the granules of human teeth particles showed that with increasing pressure, mercury penetrated into the increasingly amorphous pores. 

Pore size distribution curves must be interpreted, a technique in which it is important to specify the size range of the measured pores. The size of these spaces depends on particle size, number, and shape, as well as the distribution of particle sizes. A big peak is related to a big particle (47.2 µm), corresponding to the intrusion of mercury into the interparticle spaces. The cumulative curve denoted an intrusion into the pores of between 219 µm and 38.2 µm, followed by a plateau after 38.2 µm, where no intrusion was detected. The initial rise of the curve mostly corresponded to the filling of the spaces between the particles, whereas the later stage of rising was related to the pores within the individual particles. The intraparticle pore range was more obvious, in which one small peak at 0.0053 µm was clearly visible. 

**Figure 5:**
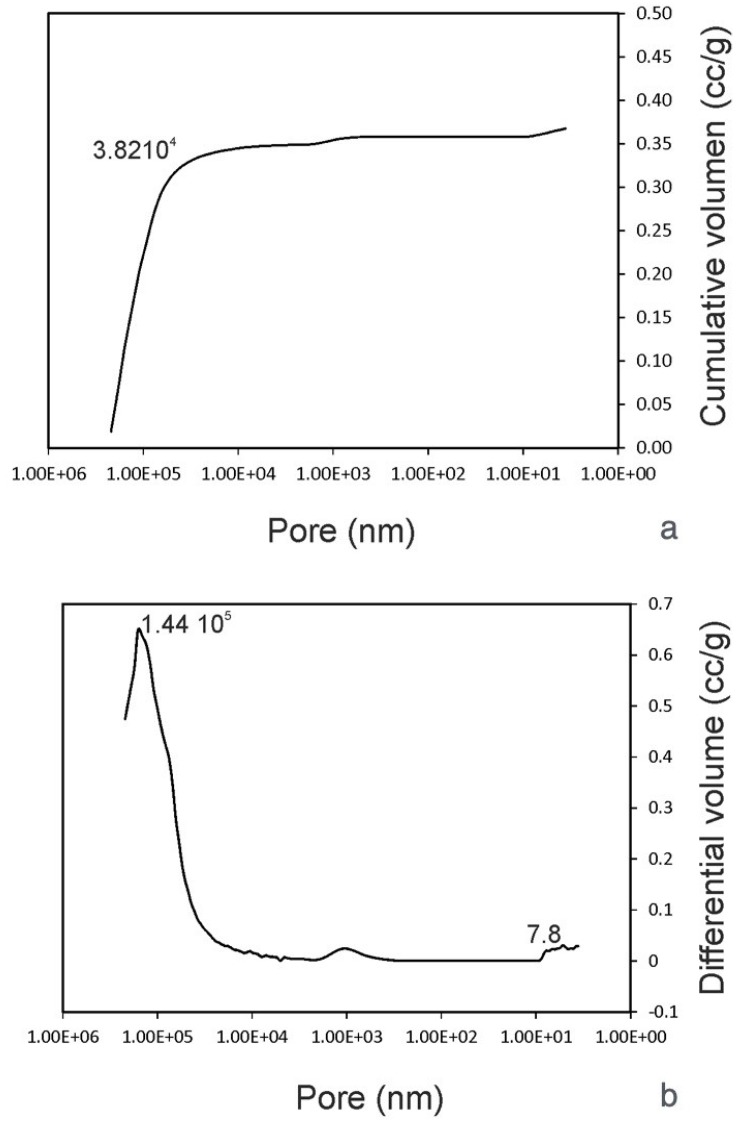
Results obtained by the helium gas pycnometer evaluating the interparticle and intraparticle porosity of the teeth grafts. (**a**) pore volume; (**b**) comparative volume pores.

These results were in accordance with the helium gas pycnometer (Table 3), in which 44.48% porosity corresponded to interparticle spaces and 2.533% corresponded to intraparticle porosity (Figure 5). 

The results of the SEM–EDX evaluation are shown in Table 4 (mean and standard deviation): O (%) 58.91 ± 1.1; Ca (%) 22.41 ± 0.28; C (%) 13.56 ± 0.44; P (%) 11.76 ± 0.45; N (%) 7.97 ± 0.21; Mg (%) 1.36 ± 0.18; and Na (%) 0.74 ± 0.45. 

## 4. Discussion

Bone graft materials derived from teeth with an absence of antigenicity improve bone formation and bone remodeling capabilities. A wide range of bone graft materials are available, and choosing the right one presents a challenging decision that will be dictated by the bone substitute material’s physicochemical properties in relation to the type of defect and the main purpose of the procedure [20,21,22].

Bone grafts derived from teeth can be considered to be an attractive option due to their autogenous origin and favorable clinical results, which have shown that these materials offer good osteoinductive capacities. Nevertheless, they pose some risk of viral infection and are limited in quantity, while most of the synthetic materials offer osteoconductive competence and can be supplied in unlimited quantities [23,24,25].

The SEM micrographs provided information about the morphology of the crushed tooth particulate, which presented no critical defect and a homogeneous microstructure with aggregates of high density. 

In mercury porosimetry analysis, the inter and intraparticle pore distinction is not always clear. The information provided by pore size distribution curves must be interpreted, a technique in which the size range of the measured pores is of fundamental importance. In the present study, the tooth particles consisted of a highly porous network with an average pore size of 0.431 ± 0.213 µm. The total porosity of the samples analyzed had an average of 54.868%, which is comparable to replacement biomaterials of different origins and with the most useful ones, which are around 60%. As research has demonstrated, the degree of porosity and its disposition directly influences the biological behavior of biomaterial grafts. In addition, there is a direct relationship between these parameters and resorption rates [26,27].

EDX was used to determine the elemental composition of the dentin particulate, obtaining a Ca/P ratio of 1.67 ± 0.09, which is similar to that of synthetic HA. The presence of traces of magnesium was also observed, known to be the impurity in calcium phosphate as a raw material. The composition of the samples determined by quantitative analysis at different points of the sample surfaces showed the presence of Ca, P, and O. 

Although demineralized dentin exhibits matrix-derived growth and differentiating factors for effective osteogenesis, the newly formed bone that is generated and the residual demineralized dentin is too weak to allow adequate implant anchorage. However, the use of the Smart Dentin Grinder Machine enables us to prepare a natural biomaterial from freshly extracted autologous teeth in the form of a bacteria-free particulate for immediate use as an autogenous graft biomaterial in a single surgical session. Teeth and mandibular/maxillary bone have a high level of similarity with dentin, both presenting similar chemical structures and composition in organic, protein, and mineral phases. For this reason, our research team (in light of our own findings and those of other investigations) proposes that non-functional extracted teeth or periodontally involved teeth should no longer be discarded [28]. Extracted teeth can be ground to produce an autogenous dentin particulate within 15 min of extraction and can then be grafted into the post-extraction alveoli. In this way, the patient’s own extracted tooth acts as a clinically useful bone graft material that offers all the advantages of autogenous bone due to the similarity of composition between bone and dentin. The particulate tooth material provides excellent biocompatibility without eliciting an immune response or a foreign material reaction or infection after it is used. In addition, it has osteoinduction, osteoconduction, and progressive substitution capabilities, and it can be worked into various sizes and shapes [28]. Moreover, some patients refuse allografts or xenografts on the basis of their origins—a problem that this technique overcomes. 

## 5. Conclusions

Autogenous tooth particulate biomaterial made from human extracted teeth may be considered a potential material for bone regeneration due to its chemical composition and the high quantity of material obtained from each tooth. After grinding the teeth, the resulting material increases in volume by up to three times, so that two extracted mandibular lateral incisors teeth will provide a sufficient amount of material to fill four empty mandibular alveoli. The tooth particles present intra- and extra-porosity up to 44.48% after pycnometer evaluation in order to increase the blood supply and support slow resorption of the grafted material, which will support healing and replacement resorption to achieve lamellar bone. After SEM–EDX evaluation, it appears that calcium and phosphates are still present within the collagen components even after the particle cleaning procedures that are conducted before use.

## Figures and Tables

**Table 1:** Descriptive test of a mean and standard deviation of each human tooth length, width, and weight of 100 teeth.

Human Teeth	Mean length ± SD (mm)	Mean width ± SD (mm)	Mean weight ± SD (gr)
Upper central incisor	6.5 ± 0.2	1.2 ± 0.6	1.3 ± 0.9
Upper lateral incisor	5.9 ± 0.4	0.9 ± 0.1	0.9 ± 0.5
Upper canine	7.1 ± 1.2	1.3 ± 0.3	1.4 ± 1.1
Upper premolar	5.6 ± 0.6	0.9 ± 0.4	1.4 ± 0.2
Upper molar	7.8 ± 0.9	1.5 ± 0.3	1.9 ± 1.1
Lower central incisor	5.2 ± 0.8	1.2 ± 0.1	0.7 ± 0.2
Lower lateral incisor	5.1 ± 0.4	1.1 ± 0.2	0.6 ± 0.7
Lower canine	6.9 ± 0.5	1.2 ± 0.7	1,2 ± 0.6
Lower premolar	6.1 ± 0.7	1.3 ± 0.6	1.4 ± 0.2
Lower molar	6.9 ± 0.2	2.1 ± 0.7	2.2 ± 1.1

**Table 2:** Comparison of the weight and volume of the human extracted teeth after grinding.

Mineralized Human Particulated Dentin Graft									
Weight after extraction	0.25 gr	0.50 gr	1.0 gr	2.0 gr	3 gr	4 gr	5 gr	6.gr	7gr
Volume after grinding	0.75 cc	1.51 cc	3.10 cc	6.11 cc	9.12 cc	12.7 cc	15.62 cc	18.21 cc	21.74 cc

**Table 3:** Mercury-intruded volume, mode (most frequent diameter) of intraparticle pores, total porosity, and interparticle porosity. (a) 1 µm < pores < 220 µm; (b) pores < 1 um.

Human Teeth	Intruded Volume (cc/g)	Total Porosity (%)	Intraparticle Porosity (%) a	Interparticle Porosity (%) b
Upper central incisor	0.321	48.31	32.13	45.78
Upper lateral incisor	0.236	44.89	33.29	44.27
Upper canine	0.456	59.87	38.78	47.81
Upper premolar	0.562	58.20	33.29	39.76
Upper molar	0.786	67.98	36.87	45.71
Lower central incisor	0.145	42.17	31.89	45.99
Lower lateral incisor	0.164	41.74	31.78	42.29
Lower canine	0.472	61.87	33.34	46.32
Lower premolar	0.501	56.98	37.65	47.22
Lower molar	0.672	66.67	38.42	48.24
Mean ± Sd	0.431 ± 0.213	54.868 ± 9.871	34.745 ± 2.841	45.339 ± 2.610

**Table 4:** Scanning electron microscopy–energy dispersive X-ray (SEM–EDX) evaluation of each crushed tooth’s chemical composition.

Human Teeth	0 (%)	Ca (%)	C (%)	P (%)	N (%)	Mg (%)	Na (%)
Upper central incisor	57.39 ± 0.11	23.78 ± 0.31	15.48 ± 0.12	9.53 ± 0.12	4.89 ± 0.11	0.96 ± 0.11	0.56 ± 0.13
Upper lateral incisor	51.38 ± 0.42	22.41 ± 0.28	14.29 ± 0.22	8.42 ± 0.11	4.07 ± 0.44	0.72 ± 0.17	0.44 ± 0.35
Upper canine	58.91 ± 1.1	24.89 ± 0.46	16.75 ± 0.23	10.23 ± 0.52	5.08 ± 0.32	0.98 ± 0.82	0.67 ± 1.8
Upper premolar	57.99 ± 0.22	24.56 ± 0.11	16.98 ± 1.87	10.55 ± 0.14	6.87 ± 0.24	1.36 ± 0.18	0.71 ± 0.23
Upper molar	61.27 ± 0.28	25.87 ± 0.67	17.39 ± 0.26	11.76 ± 0.45	7.97 ± 0.21	1.79 ± 0.22	0.74 ± 0.45
Lower central incisor	49.87 ± 0.33	21.11 ± 0.72	13.56 ± 0.44	7.82 ± 0.12	4.01 ± 0.66	0.77 ± 0.14	0.88 ± 0.56
Lower lateral incisor	48.66 ± 0.26	20.78 ± 0.65	13.11 ± 0.27	7.43 ± 0.54	3.99 ± 0.81	0.69 ± 0.36	0.48 ± 0.12
Lower canine	52.19 ± 0.15	24.56 ± 0.77	16.21 ± 0.98	9.68 ± 0.78	4.67 ± 0.81	0.97 ± 0.26	0.66 ± 0.24
Lower premolar	53.46 ± 0.23	24.82 ± 0.12	16.34 ± 0.29	10.23 ± 0.56	5.47 ± 0.54	1.06 ± 0.31	0.79 ± 0.33
Lower molar	57.82 ± 0.45	25.65 ± 0.38	17.13 ± 0.31	10.98 ± 0.33	6.03 ± 0.16	1.45 ± 0.24	0.82 ± 0.12
